# Prognostic Significance of Plasma Lactate Levels in Postcardiotomy Patients

**DOI:** 10.1016/j.jacadv.2026.103158

**Published:** 2026-08-18

**Authors:** B. Ufuk Baldan, Mariëlle C. van de Veerdonk, Pieter-Roel Tuinman, Susanne Eberl, José P.S. Henriques, Robert J.M. Klautz, Patrick Klein

**Affiliations:** a Department of Cardiothoracic Surgery, Amsterdam University Medical Center, Amsterdam Cardiovascular Sciences, University of Amsterdam, Amsterdam, the Netherlands; b Department of Cardiothoracic Surgery, Leiden University Medical Center, University of Leiden, Leiden, the Netherlands; c Department of Cardiology, Amsterdam University Medical Center, Amsterdam Cardiovascular Sciences, University of Amsterdam, Amsterdam, the Netherlands; d Department of Intensive Care, Amsterdam University Medical Center, Amsterdam Cardiovascular Sciences, University of Amsterdam, Amsterdam, the Netherlands; e Department of Anesthesiology, Amsterdam Univaersity Medical Center, Amsterdam Cardiovascular Sciences, University of Amsterdam, Amsterdam, the Netherlands

**Keywords:** cardiac surgery, cardiogenic shock, plasma lactate, postcardiotomy, SCAI

## Abstract

**Background:**

Although plasma lactate levels have established prognostic value in cardiac critical care, current shock thresholds are largely derived from nonsurgical cardiogenic shock populations and may not apply to postcardiotomy patients.

**Objectives:**

The objectives of the study were to examine the association between plasma lactate levels during the first 5 postoperative days (PODs) and in-hospital mortality in postcardiotomy patients, and to compare the discriminative performance with the SCAI (Society of Cardiovascular Angiography and Interventions) SHOCK classification.

**Methods:**

This retrospective, multicenter study included consecutive adult postcardiotomy patients from 2 academic centers between 2015 and 2024. Plasma lactate levels were measured every 6 hours, with levels of >2.0 mmol/L considered elevated. The primary endpoint was in-hospital mortality.

**Results:**

Among the 8,519 patients, the median age was 68 years (Q1-Q3: 59-74 years) and 72% were males. Overall in-hospital mortality was 2%. The highest levels of plasma lactate were observed on POD 2, with 46% of patients having elevated plasma lactate, declining thereafter. In-hospital mortality increased significantly at plasma lactate levels ≥5.0 mmol/L. Every 1 mmol/L increase in plasma lactate was independently associated with higher in-hospital mortality risk after multivariable adjustment. The SCAI SHOCK classification demonstrated higher discriminative performance to predict in-hospital mortality compared to plasma lactate on POD 1 (area under the curve: 0.81 vs 0.75; *P* = 0.050), whereas values were comparable on the following PODs.

**Conclusions:**

Elevated plasma lactate levels were common after cardiac surgery and independently associated with in-hospital mortality, with risk rising exponentially from ≥5.0 mmol/L. No statistically significant differences in discriminative performance between plasma lactate, SCAI SHOCK classification, and EuroSCORE (European System for Cardiac Operative Risk Evaluation) II were observed.

Plasma lactate levels are known to have prognostic value in cardiac critical care and after cardiac surgery.^1^, ^2^, ^3^ Yet, current classifications of cardiogenic shock (CS) incorporate plasma lactate thresholds derived mainly from nonsurgical populations, such as acute myocardial infarction (AMI)-related and decompensated heart failure-related CS. These thresholds may not adequately reflect the pathophysiology of postcardiotomy shock (PCS).^2^^,^^4^, ^5^, ^6^ Cardiopulmonary bypass, myocardial ischemia-reperfusion, systemic cooling and rewarming, and perioperative fluid and transfusion strategies are all inherent to PCS and may contribute to plasma lactate accumulation and modify its prognostic implications.^2^^,^^3^^,^^7^^,^^8^

The SCAI (Society for Cardiovascular Angiography and Interventions) SHOCK stage classification, which incorporates plasma lactate levels, has been validated in PCS populations.^4^^,^^9^^,^^10^ However, the relationship between this clinical staging system and plasma lactate dynamics in postcardiotomy patients during the early postoperative period remains incompletely understood.

Our primary objective was to investigate the relationship between plasma lactate levels and in-hospital mortality in postcardiotomy patients across the first 5 postoperative days (PODs). The secondary objective was to compare the discriminative performance of plasma lactate alone compared with the SCAI SHOCK classification for predicting in-hospital mortality.

## Methods

### Ethics statement

The study was reviewed and approved by the Medical Research Ethics Committee (Amsterdam, the Netherlands) (2024.0184) and conducted in accordance with the Declaration of Helsinki.

### Design

This retrospective, observational, multicenter cohort study included all consecutive adult patients who underwent cardiac surgery between January 2015 and June 2024 at 2 academic hospitals in the Netherlands (VU University Medical Center and Amsterdam University Medical Center).

### Patients

Patients were eligible for inclusion if they met the following criteria: 1) postcardiotomy patients; 2) ≥18 years of age; and 3) patient body weight ≥40 kg.

Exclusion criteria were: 1) bail-out procedure before surgery, such as rescue percutaneous coronary intervention or mechanical circulatory support, including venoarterial extracorporeal life support, Impella and intra-aortic balloon pump; 2) isolated surgery for atrial fibrillation; 3) no measurement of plasma lactate level on POD 1; and 4) need for emergent surgery (within 24 hours after diagnosis). A detailed flowchart of patient inclusion and exclusion is provided in Supplemental Figure 1.

### Data collection

Patient data were extracted from the electronic health record system Epic (Epic Systems Corporation, Verona, Wisconsin, USA) and included demographics, medical history, vital signs, surgical characteristics, and preoperative and postoperative laboratory values, and postoperative outcomes including morbidity and in-hospital mortality. Data were collected daily from POD 1 through 5. All information was stored in a centralized database, and variable definitions adhered to standardized criteria consistent with those outlined by the NHR (Dutch Heart Registry)^11^ and summarized in Supplemental Text 1. The 2022 SCAI SHOCK classification system, as updated by Cardiogenic Shock Working Group and modified by Kapur et al,^12^ was used to stratify the patients on POD 1.^4^ Staging, as shown in Supplemental Figure 2, incorporated lowest blood pressure values, highest plasma lactate levels, and number of vasoactive-inotropic agents administered. Blood pressure was measured every 3 hours for SCAI SHOCK staging.

### Plasma lactate

Plasma lactate levels were measured routinely every 6 hours during intensive care unit admission, with more frequent measurements performed when clinically indicated based on patient's hemodynamic status. For each POD, the highest recorded plasma lactate level within that 24-hour period was selected for analysis. These daily highest plasma lactate levels were compared between survivors and nonsurvivors for each POD. All patients were additionally stratified into 4 plasma lactate categories of 0 to 2 mmol/L, 2 to 5 mmol/L, 5 to 10 mmol/L, and >10 mmol/L based on their highest plasma lactate level from POD 1 through 5. These cutoffs align with the SCAI SHOCK classification.^4^^,^^12^ In-hospital mortality rates were calculated for each category to identify plasma lactate thresholds associated with increased risk for in-hospital mortality. Plasma lactate levels of >2.0 mmol/L were considered elevated. For longitudinal analyses, plasma lactate levels were additionally structured as repeated daily measurements across POD 1 through 5.

### Outcomes

The primary outcome was all-cause in-hospital mortality and its association with absolute plasma lactate levels during the first 5 PODs, assessed both descriptively by POD and longitudinally using daily plasma lactate levels. The secondary outcome was the discriminative performance of plasma lactate alone compared with SCAI SHOCK classification for predicting in-hospital mortality.

### Statistical analysis

The study population was summarized using descriptive statistics. Continuous variables are presented as mean ± SD or median (Q1-Q3) and were tested for normality using the Shapiro-Wilk test. Comparisons were made using the independent Student's *t-*test or Kruskal-Wallis test. Categorical variables are reported as counts and percentages and were analyzed using the chi-square test or Fisher exact test, as appropriate.

Postoperative plasma lactate trajectories were analyzed using a mixed-effects model with POD and a quadratic POD term as fixed effects and random intercepts and slopes per patient.

The association between plasma lactate and in-hospital mortality was assessed using time-dependent Cox proportional hazards models, with plasma lactate as a time-varying covariate updated daily. A univariable model was fitted first. The multivariable model adjusted for age, EuroSCORE (European System for Cardiac Operative Risk Evaluation) II, left ventricular ejection fraction (LVEF) (>50%, 41%-50%, ≤40%), procedural complexity (isolated coronary artery bypass grafting, isolated non–coronary artery bypass grafting procedure, 2 procedures, and ≥3 procedures), and (concomitant) mitral, aortic, tricuspid valve, and aortic surgery. Results are reported as HRs with 95% CIs. For the multivariable time-dependent Cox models with daily plasma lactate levels, we checked the proportional hazards assumption using the Schoenfeld test. We inspected the residual plots for each covariate and used an overall test to see whether the model showed important deviations from proportional hazards.

Because discharge alive is a competing event for in-hospital mortality, we additionally fitted a Fine-Gray model with in-hospital mortality and discharge alive as the competing event. The model was adjusted for the same covariates as in the multivariable Cox models.

Receiver-operating characteristic curve analysis with area under the curve (AUC) calculation was performed to compare the SCAI SHOCK classification with absolute plasma lactate levels and EuroSCORE II. Paired DeLong tests between SCAI SHOCK classifications were applied to compare AUCs between models at each POD. Given repeated pairwise AUC comparisons across PODs, Holm-Bonferroni correction was applied to DeLong test *P* values for each comparison. AUC estimates are reported with 95% CI. In addition, model calibration was assessed using Brier scores and calibration plots, comparing observed in-hospital mortality with model-predicted mortality across 10 equally sized groups of increasing predicted risk (deciles of predicted probability). Statistical significance was set at *P* < 0.05. Missing data were handled using a complete-case approach. Patients with missing values for covariates included in the multivariable Cox models were excluded from the respective analysis. Analyses were performed using R, version 4.3.2 and 4.6.0 (RStudio, R Foundation for Statistical Computing).

## Results

### Study population

The study included 8,519 patients with a median age of 68 years (Q1-Q3: 59-74 years), and 72% were males, as presented in Table 1. Most procedures were elective (61%). Nine percent of the patients had undergone prior cardiac surgery. Preoperatively, left ventricular function was preserved in 72%. Pulmonary hypertension was moderate in 15% and severe in 1%. Most patients underwent 1 procedure (71%), whereas 21% had 2 procedures and 8% had 3 or more. Overall, in-hospital mortality was 2% (n = 167). Among those who died, 2% died on POD 1, 11% on POD 2, 9% on POD 3, 8% on POD 4, and 7% on POD 5.

**Table 1:** Baseline Characteristics, Surgical Data and Outcome of Surviving and Nonsurviving Postcardiotomy Patients on POD 1

	N	Survivors (n = 8,352)	Nonsurvivors (n = 167)	*P* Value
Male	72	72	59	0.001
Age, y	68 (59-74)	68 (59-74)	72 (63-76)	<0.001
BMI	27 (24-29)	27 (24-29)	28 (24-32)	0.001
NYHA functional class				
I	42	42	26	<0.001
II	29	28	16	<0.001
III	24	24	29	0.09
IV	5	6	30	<0.001
Diabetes mellitus	19	19	19	0.97
Previous cardiac surgery	9	9	25	<0.001
Endocarditis	4	3	21	<0.001
LVEF				
Good	72	72	56	<0.001
Moderate	0	0.3	0	1.00
Poor	28	28	44	<0.001
Estimated sPAP				
Normal	84	85	67	<0.001
Moderate	15	14	29	<0.001
High	1	1	3	0.009
Urgency				
Elective	61	61	26	<0.001
Urgent	39	39	39	0.4
EuroSCORE II, %	2 (1-4)	2 (1-4)	7 (4-16)	<0.001
Procedures				
1 procedure, isolated CABG	42	41	21	<0.001
1 procedure, no CABG	30	30	26	0.4
2 procedures	21	21	29	0.003
≥3 procedures	8	8	24	<0.001
Intensive care stay, d	1 (1-2)	1 (1-2)	3 (1-8)	<0.001
Hospital stay, d	7 (6-10)	7 (6-10)	11 (5-24)	<0.001
SCAI SHOCK stage				
A	44	43	5	<0.001
B	21	21	6	<0.001
C	26	27	27	1.00
D	7	8	25	<0.001
E	2	2	31	<0.001
ECLS				
Intraoperatively	0.2	0.2	5	<0.001
Postoperatively	0.1	0.1	7	<0.001
CVA with permanent, new-onset, postoperative neurological dysfunction	1	1	9	<0.001
Renal failure	2	2	18	<0.001
Rhythm problem	11	11	5	0.04
Respiratory insufficiency	1	1	3	<0.001
Vasopressor				
I	27	27	64	<0.001
II	1	1	6	<0.001
Inotropes				
I	10	10	33	<0.001
II	1	1	11	<0.001
III	0	0	3	<0.001
Hemoglobin, POD 1, mmol/L	8 (7-8)	8 (7-8)	7 (6-8)	<0.001
Plasma lactate, POD 1, mmol/L	2 (1-2)	2 (1-2)	3 (2-7)	<0.001
CK-MB, POD1, μg/L	25 (16-41)	25 (16-41)	41 (22-88)	<0.001
Creatinine, POD 1, μmol/L	86 (74-98)	83 (71-99)	101 (81-132)	<0.001

Values are % or median (Q1-Q3), unless otherwise indicated.

For all definitions of variables, the standardized criteria outlined by the NHR (Dutch Heart Registry) are used (20), and some of them are summarized in text.

BMI = body mass index; CABG = coronary artery bypass grafting; CK-MB = creatine kinase-myocardial band; CVA = cerebral vascular accident; ECLS = extra corporeal life support; EuroSCORE = European System for Cardiac Operative Risk Evaluation; LVEF = left ventricular ejection fraction; POD = postoperative day; SCAI = Society for Cardiovascular Angiography and Interventions; sPAP = systolic pulmonary artery pressure.

### Absolute plasma lactate levels and in-hospital mortality

**Figure 1:**
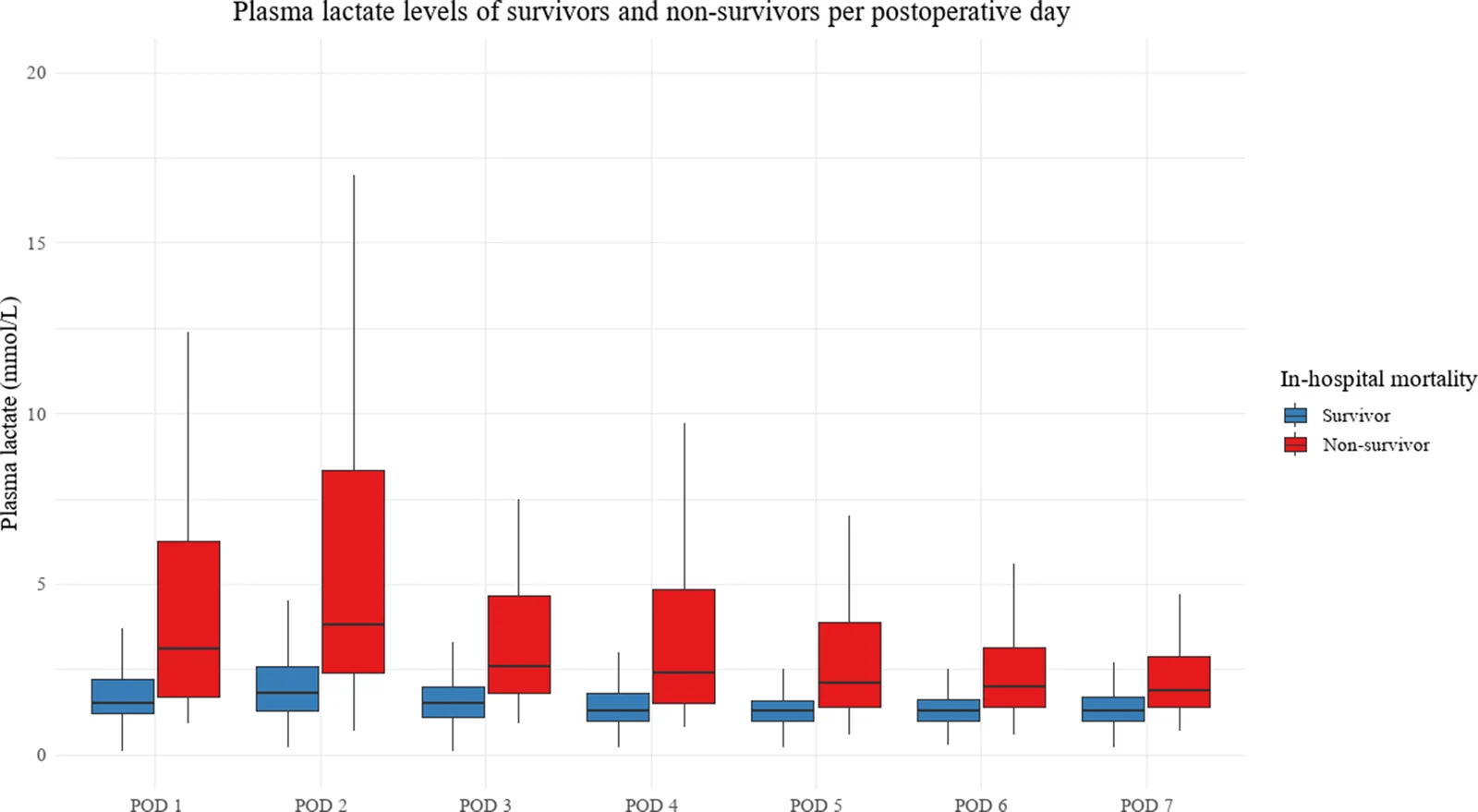
Daily Absolute Maximum Plasma Lactate Levels POD = postoperative day.

**Figure 2:**
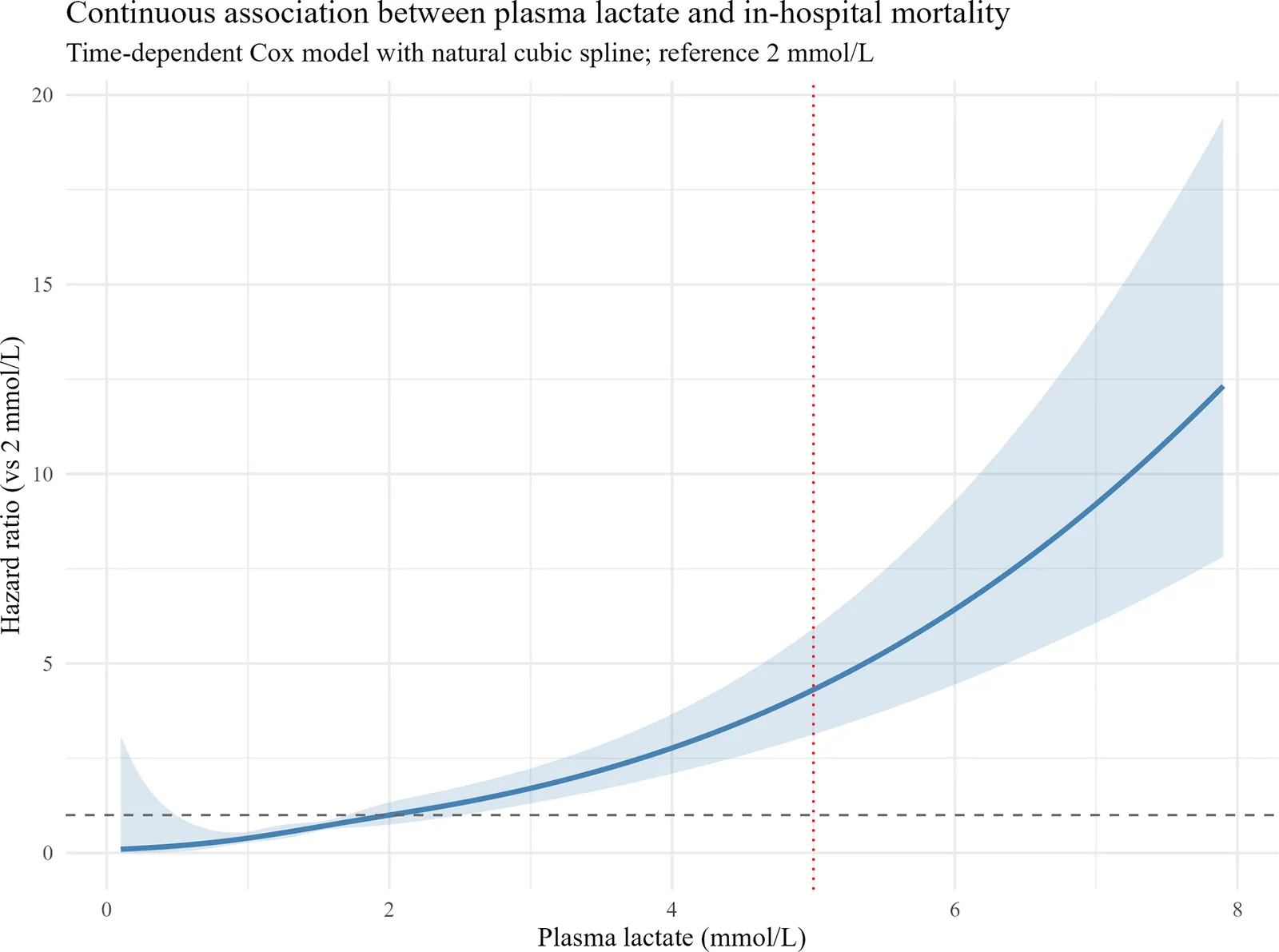
Continuous Association Between Plasma Lactate and In-Hospital Mortality The adjusted HR (aHR) was aHR: 1.7 (95% CI: 1.2-2.5) at 3.0 mmol/L, aHR: 2.8 (95% CI: 1.9-4.1) at 4.0 mmol/L, aHR: 4.3 (95% CI: 2.8-6.6) at 5.0 mmol/L, aHR: 6.4 (95% CI: 4.0-10.3) at 6.0 mmol/L, aHR: 12.7 (95% CI: 7.4-21.8) at 8.0 mmol/L, and aHR: 21.7 (95% CI: 12.0-39.4) at 10.0 mmol/L.

The median plasma lactate levels in the overall cohort per POD are reported in Supplemental Table 1. Throughout POD 1 through 5, nonsurvivors had consistently higher plasma lactate levels compared to survivors (Figure 1). Plasma lactate levels ranged widely within both groups. Analysis of predefined plasma lactate categories demonstrated a progressive increase in in-hospital mortality risk across all plasma lactate ranges. Among patients with plasma lactate levels of 2 to 5 mmol/L on POD 1, in-hospital mortality was 3%, as compared with 1% among those with levels below 2 mmol/L. The in-hospital mortality risk increased substantially at plasma lactate levels ≥5.0 mmol/L, with 18% in-hospital mortality in the 5 to 10 mmol/L category and 41% in-hospital mortality in the >10 mmol/L category (Table 2). Similar patterns were observed across POD 2 through 5 (Supplemental Tables 3-6). To evaluate whether this association was driven by an arbitrary threshold, we modeled daily plasma lactate as a continuous predictor as shown in Figure 2. This analysis shows a progressively higher risk from 5.0 mmol/L.

**Table 2:** Distribution of Patients Across Predefined Plasma Lactate Categories (0-2 mmol/L, 2-5 mmol/L, 5-10 mmol/L, and >10 mmol/L) With Corresponding Survival and In-Hospital Mortality per Category on POD 1

	Survivors (n = 8,352)	Nonsurvivors (n = 167)	*P* Value	OR (95% CI)	*P* Value
0-2 mmol/L	5,676 (99)	49 (1)	<0.001	0.2 (0.1-0.3)	<0.001
2-5 mmol/L	2,477 (98)	63 (3)	<0.001	1.4 (1.0-2.0)	<0.001
5-10 mmol/L	173 (82)	37 (18)	<0.001	13.4 (8.8-20.2)	<0.001
>10 mmol/L	26 (59)	18 (41)	<0.001	38.6 (19.5-74.9)	<0.001

### Longitudinal plasma lactate trajectories and in-hospital mortality risk

Higher plasma lactate levels were strongly associated with in-hospital mortality across POD 1 through 5. After adjustment for age, EuroSCORE II, LVEF, procedural complexity, and concomitant surgery, each 1 mmol/L increase in plasma lactate was associated with a 1.2- to 1.5-fold higher hazard of in-hospital mortality (Figure 2). The overall Schoenfeld test did not suggest violations of the proportional hazards assumption (*P* = 0.15). For plasma lactate itself, the test indicated that its effect changed over time and is consistent with the POD-specific adjusted HRs reported in Figure 2 (*P* = 0.001).

In the mixed-effects analysis, predicted mean plasma lactate was 2.0 mmol/L on POD 1, peaked at 2.1 mmol/L on POD 2, and then declined to 1.9, 1.5, and 0.9 mmol/L on POD 3 through 5. Patients with higher initial levels showed steeper declines but remained elevated over time. In time-dependent Cox models, higher plasma lactate was independently associated with in-hospital mortality after multivariable adjustment.

In the competing-risk analysis, higher plasma lactate on POD 1 remained strongly associated with in-hospital mortality. The HR was 1.5 (95% CI: 1.4-1.5) per 1 mmol/L increase in plasma lactate in the univariable model and 1.4 (95% CI: 1.3-1.5) after multivariable adjustment.

### Elevated plasma lactate levels

The proportion of patients with elevated plasma lactate increased from 33% on POD 1 to 46% on POD 2 and decreased on subsequent days (Table 3). Among patients with elevated plasma lactate levels, the percentage of nonsurvivors increased progressively from POD 1 (4%) to POD 5 (30%). Survival rates decreased progressively among those who maintained elevated plasma lactate levels on subsequent days.

**Table 3:** Percentages of Elevated Plasma Lactate Levels (>2 mmol/L) of Total Cohort per POD (1 to 5) and Percentages Nonsurvivors of the Patients With Elevated Levels

	Elevated Plasma Lactate Level (>2 mmol/L)	Nonsurvivors of the Elevated Plasma Lactate Group
POD 1 (n = 8,519)	2,794 (33)	118 (4)
POD 2 (n = 8,055)	3,678 (46)	126 (3)
POD 3 (n = 2,819)	831 (30)	86 (10)
POD 4 (n = 1,402)	326 (23)	65 (20)
POD 5 (n = 862)	167 (19)	50 (30)

Values are n(%).

Abbreviation as in Table 1.

Univariate analysis identified several significant risk factors associated with elevated plasma lactate on POD 1 (Supplemental Table 2). The strongest associations were observed for preoperative NYHA functional class IV (OR: 2.2; 95% CI: 1.8-2.7; *P* < 0.001), mitral valve surgery (OR: 2.0; 95% CI: 1.8-2.3; *P* < 0.001), 3 or more procedures (OR: 1.9; 95% CI: 1.6-2.2; *P* < 0.001), endocarditis (OR: 1.8; 95% CI: 1.4-2.2; *P* < 0.001), and previous cardiac surgery (OR: 1.6; 95% CI: 1.3-1.8; *P* < 0.001). Conversely, several factors were associated with lower odds of elevated plasma lactate, including elective surgery (OR: 0.7; 95% CI: 0.6-0.8; *P* < 0.001), 1 procedure (OR: 0.7; 95% CI: 0.6-0.8; *P* < 0.001), NYHA functional class II (OR: 0.8; 95% CI: 0.7-0.8; *P* < 0.001), a normal estimated pulmonary artery pressure (OR: 0.8; 95% CI: 0.7-0.9; *P* < 0.001), and good LVEF (OR: 0.9; 95% CI: 0.8-0.9; *P* = 0.003).

### The SCAI SHOCK classification

**Figure 3:**
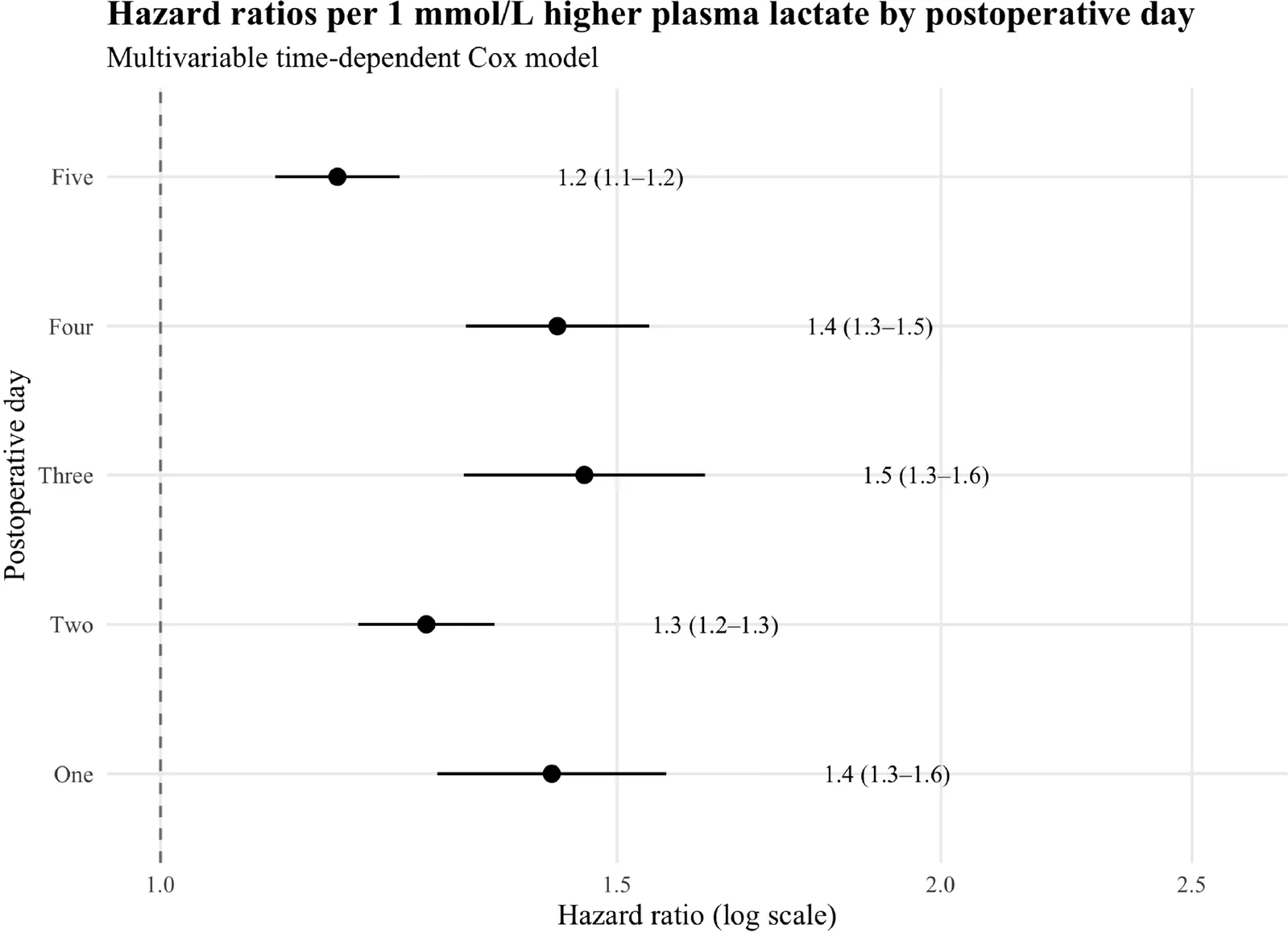
aHRs for In-Hospital Mortality The aHR per 1 mmol/L increase in plasma lactate was aHR: 1.4 (95% CI: 1.3-1.6) on POD 1, aHR: 1.3 (95% CI: 1.2-1.3) on POD 2, aHR: 1.5 (95% CI: 1.3-1.6) on POD 3, aHR: 1.4 (95% CI: 1.3-1.5) on POD 4, and aHR: 1.2 (95% CI: 1.1-1.2) on POD 5. Abbreviations as in Figures 1 and 2.

**Figure 4:**
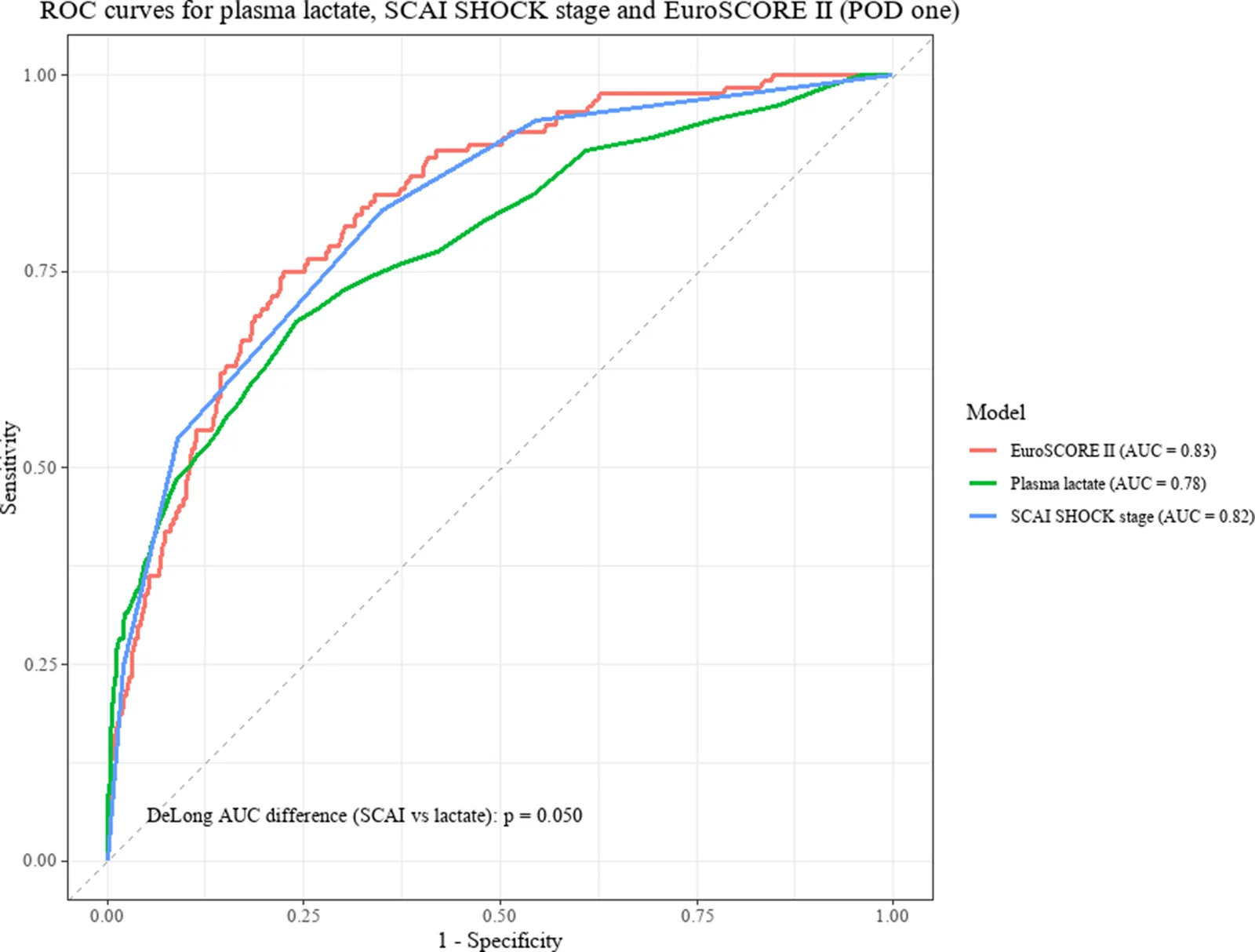
ROC: Plasma Lactate vs SCAI SHOCK on POD 1 AUC = area under the curve; EuroSCORE = European System for Cardiac Operative Risk Evaluation; NS = not significant; ROC = Receiver operating characteristic; SCAI = Society for Cardiovascular Angiography and Interventions; other abbreviation as in Figure 1.

The SCAI SHOCK stage classification showed a slightly higher discriminative performance for the prediction of in-hospital mortality on POD 1 compared to plasma lactate alone (AUC: 0.82 [95% CI: 0.79-0.86] vs 0.78 [95% CI: 0.73-0.83]; *P* = 0.050) (Figure 3). EuroSCORE II had similarly high discrimination (AUC: 0.83; 95% CI: 0.80-0.87]. On POD 2 through 5, the predictive performance was comparable between plasma lactate and SCAI SHOCK (Figure 4, Table 4, Central Illustration). EuroSCORE II showed AUC values comparable to SCAI SHOCK on POD 1 and 2, but the lowest discriminative performance from POD 3 onward (Supplemental Figures 3-7). Given the repeated measurements, a Holm-Bonferroni correction was applied, after which no differences remained statistically significant (all values of *P* ≥ 0.05). The corresponding AUC for each POD and each plasma lactate threshold within the SCAI SHOCK classification are shown in Supplemental Table 7. Brier scores for EuroSCORE II, SCAI SHOCK stage and plasma lactate were 0.0166, 0.0162, and 0.0161 on POD 1. On PODs 2 to 5, Brier scores increased modestly for all 3 models and calibration plots for each POD showed how predicted probabilities relate to observed mortality (Supplemental Table 8, Supplemental Figures 8-12).

**Central Illustration:**
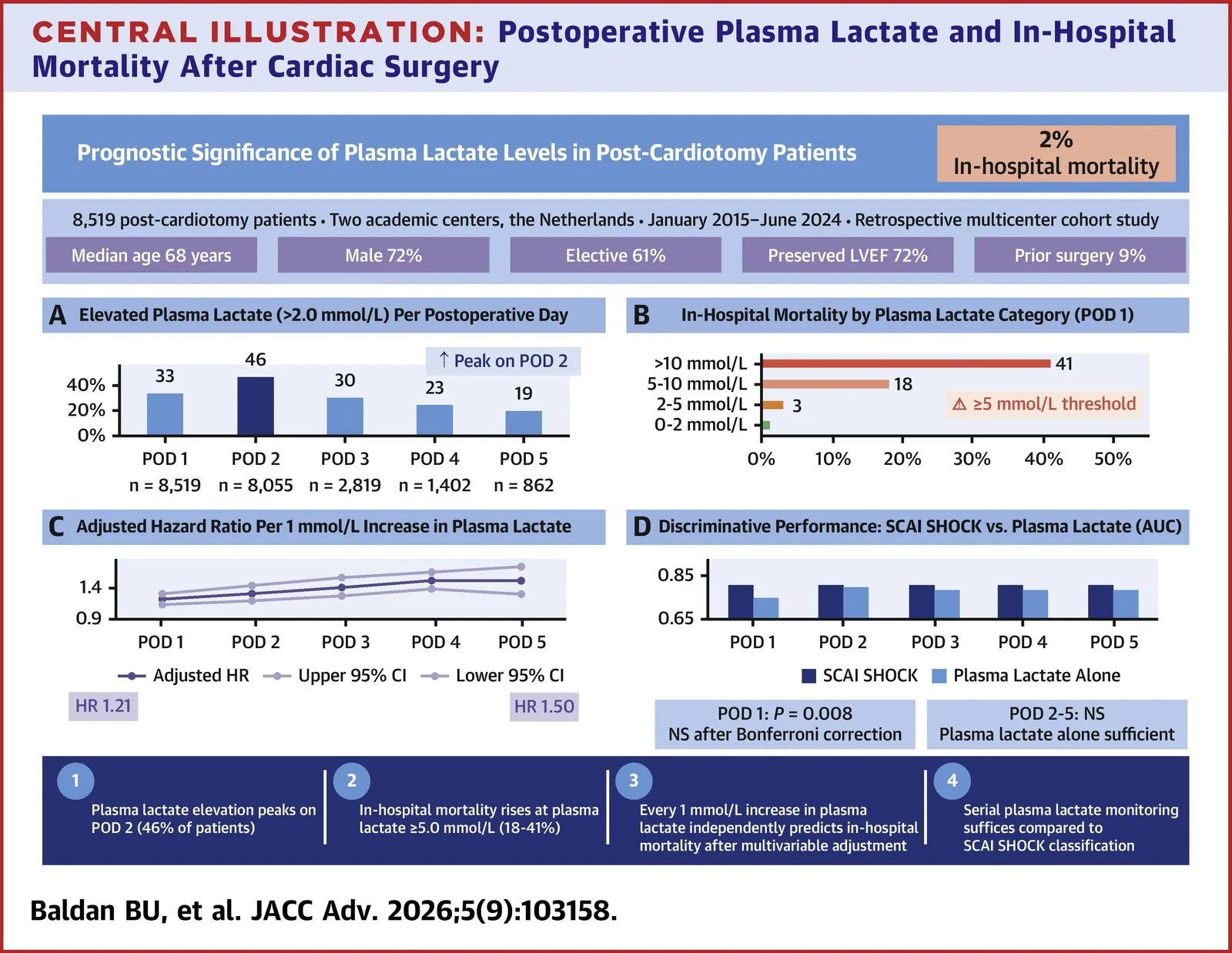
Postoperative Plasma Lactate and In-Hospital Mortality After Cardiac Surgery LVEF = left ventricular ejection fraction; NS = not significant; POD = postoperative day; SCAI = Society for Cardiovascular Angiography and Interventions.

**Table 4:** ROC Curve Analysis for Plasma Lactate Levels vs SCAI SHOCK on POD 2 Through 5

	Plasma Lactate	SCAI SHOCK Stage	*P* Value
POD 2: AUC	0.80	0.81	0.15
POD 3: AUC	0.79	0.81	0.05
POD 4: AUC	0.78	0.79	0.25
POD 5: AUC	0.77	0.80	0.07

AUC = area under the curve; ROC = receiver-operating characteristic; other abbreviations as in Table 1.

## Discussion

This retrospective, observational, multicenter study of 8,519 postcardiotomy patients on absolute plasma lactate levels yielded 5 major findings: 1) elevated plasma lactate level were common during the first 2 PODs after cardiac surgery; 2) both plasma lactate levels and the proportion of patients with elevated plasma lactate were highest on POD 2, followed by a progressive decline; 3) nonsurvivors consistently demonstrated higher plasma lactate levels than survivors across all PODs; 4) the prognostic value of elevated plasma lactate for in-hospital mortality increased exponentially at ≥5.0 mmol/L, and every 1 mmol/L increase in plasma lactate was independently associated with higher in-hospital mortality risk after multivariable adjustment; and 5) SCAI SHOCK staging provided superior early risk stratification compared to plasma lactate alone on POD 1, whereas from POD 2 onward their discriminative performance was comparable.

Our findings extend previous reports on plasma lactate in postcardiotomy patients. Although Maillet et al^8^ observed a progressive rise in plasma lactate over a 24-hour observation period and Hajjar et al^7^ demonstrated that initial plasma lactate levels predict complications, our longitudinal analysis of 5 consecutive PODs provided a more comprehensive trajectory of plasma lactate levels over time. Although the prognostic value of plasma lactate in critically ill patients is well established, its temporal dynamics and clinically relevant thresholds in postcardiotomy patients specifically have remained poorly defined. The peak on POD 2 has direct clinical relevance. It reframes early postoperative elevated plasma lactate as a potentially physiological response rather than an immediate marker of refractory shock.

In our cohort, plasma lactate levels showed a transient peak on POD 2, with subsequent decline in most patients. This initial postoperative rise may represent a normal physiological response rather than persistent shock, potentially explained by vasoplegic syndrome and myocardial stunning.^2^^,^^3^^,^^6^^,^^7^ This nonlinear pattern was also captured in the longitudinal mixed-effects trajectory analysis. The observed peak and subsequent decline should, however, be interpreted with caution, as 13% of nonsurvivors died before POD 2 and were therefore no longer included in the analysis. In addition, multiple factors beyond tissue hypoperfusion contribute to plasma lactate dynamics, including impaired hepatic and renal clearance, inflammatory-mediated metabolic alterations, mechanical ventilation, and mitochondrial dysfunction from ischemia-reperfusion injury.^1^, ^2^, ^3^^,^^7^

We found that plasma lactate levels ≥5.0 mmol/L were associated with increased in-hospital mortality and rose progressively across increasing plasma lactate categories. This is consistent with thresholds previously reported in AMI-CS cohorts.^4^^,^^6^^,^^13^^,^^14^ However, the temporal pattern differed. In AMI-CS, plasma lactate rises acutely within hours of presentation, whereas in our cohort levels peaked on POD 2 rather than immediately following surgery.^2^, ^3^, ^4^^,^^6^ This may suggest different underlying mechanisms despite comparable prognostic thresholds.^2^, ^3^, ^4^^,^^6^ Beyond absolute thresholds, plasma lactate trajectories reflect both disease severity and treatment adequacy and response, with persistently elevated levels potentially indicating either refractory pathophysiology or suboptimal intervention.^3^^,^^6^^,^^13^ In this regard, whether plasma lactate normalizes or persists over time may carry greater prognostic relevance than any single time point measurement.^13^, ^14^, ^15^

The association between plasma lactate and in-hospital mortality strengthened across PODs. Each analysis was restricted to surviving ICU patients. Rising plasma lactate on later PODs may reflect progressive low cardiac output, end-organ hypoperfusion, or refractory CS. However, survivor bias is a major concern in this analysis, as the cohort becomes increasingly selected and high-risk over time, and findings should therefore be interpreted with caution.

Our findings suggest that, in practice, it may matter little whether plasma lactate, SCAI SHOCK stage, or EuroSCORE II is used for postoperative risk stratification, because these 3 predictors showed largely similar discriminative performance across PODs 1 through 5. No statistically significant AUC differences remained after adjustment for multiple comparisons. At the same time, it should be emphasized that plasma lactate is directly incorporated into the SCAI SHOCK classification, so these predictors are not independent and any apparent differences should be interpreted in light of this dependency. Taken together, these data indicate that serial plasma lactate measurements may offer prognostic information comparable to SCAI SHOCK staging for both early and later postoperative risk stratification. In this context, systematic use of plasma lactate may provide a pragmatic and easily implementable approach to postoperative risk assessment, whereas SCAI SHOCK staging can still add clinical nuance through blood pressure and vasoactive support. In contrast, in nonsurgical CS cohorts SCAI SHOCK staging has shown prognostic superiority over plasma lactate alone.^2^, ^3^, ^4^, ^5^^,^^12^ However, these observations should be regarded as hypothesis-generating rather than practice-defining.

### Study strengths and limitations

This study has several strengths. The large sample size of 8,519 patients from 2 Dutch academic centers provides substantial statistical power. Measuring plasma lactate levels across 5 PODs allowed us to describe temporal patterns in detail. The inclusion of almost all cardiac surgical procedures, excluding advanced heart failure therapies and heart transplantation, enhances the generalizability of findings to real-world clinical practice.

Several limitations must be acknowledged. The retrospective design limits causal inference and introduces potential selection bias. Missing or unmeasured variables may have introduced bias and limited generalizability, potentially resulting in suboptimal risk adjustment. Missing covariate data were limited and handled using complete-case analysis. In addition, exclusion of patients without a POD 1 plasma lactate level or those requiring emergent surgery may have selected a lower-risk population, and in-hospital mortality estimates could therefore be interpreted as conservative. Although our longitudinal mixed-effects and time-dependent modeling reduces some limitations, residual confounding and informative censoring remain possible in this retrospective design. The association of plasma lactate with in-hospital mortality varies over time, and the Cox model therefore summarizes this as an average hazard effect over POD 1 through 5. We partly addressed this by modeling plasma lactate as a daily covariate and by reporting POD-specific adjusted HRs, but some time-dependence remains. Our multivariable models included important covariates; however, other important perioperative factors such as cardiopulmonary bypass time, fluid and transfusion requirements, and vasopressor-inotropic score were not systematically captured in both centers and could not be incorporated.

Although this was a multicenter study, external validation in international, prospective cohorts is required. Selection bias may have been introduced by the exclusion criteria, as patients undergoing bail out procedures or those without early plasma lactate measurements represent clinically important high-risk or low-risk subgroups. We did not fit surgeon- and center-specific multilevel models, because case-mix and outcomes were similar and the numbers within individual surgeon-procedure groups were too small for stable analysis. Finally, although our multivariable models included important covariates, important perioperative factors such as cardiopulmonary bypass time, fluid and transfusion requirements, and vasopressor-inotropic score were not systematically captured and could not be incorporated.

## Conclusions

Elevated plasma lactate levels were common after cardiac surgery and independently associated with in-hospital mortality, with risk rising exponentially from ≥5.0 mmol/L. No statistically significant differences in discriminative performance between plasma lactate, SCAI SHOCK classification, and EuroSCORE II across PODs 1 through 5 were observed.

Perspectives **COMPETENCY IN PATIENT CARE AND PROCEDURAL SKILLS:** The observed postoperative plasma lactate trajectory, with a peak on POD 2 and subsequent decline in most patients, should inform monitoring strategies. Particular attention should be given to patients with persistently elevated or rising plasma lactate levels, as longitudinal analyses showed an independent association between higher daily plasma lactate and in-hospital mortality.

**COMPETENCY IN MEDICAL KNOWLEDGE:** Plasma lactate levels were highest on POD 2, reflecting common pathophysiology following cardiac surgery. An increase in in-hospital mortality is observed at plasma lactate levels ≥5.0 mmol/L. Repeated postoperative plasma lactate measurements provide prognostic information over time, and higher plasma lactate levels remain independently associated with in-hospital mortality after adjustment for clinical risk factors. SCAI SHOCK staging does not appear to be more informative for risk stratification compared to plasma lactate and EuroSCORE II on PODs 1 through 5.

**TRANSLATIONAL OUTLOOK:** Implementation of plasma lactate monitoring systems and automated alerts for elevated plasma lactate levels (particularly ≥5.0 mmol/L) can facilitate timely additional imaging and intervention. Serial plasma lactate monitoring may offer a pragmatic alternative to SCAI SHOCK classification for postoperative risk stratification.

## Funding support and author disclosures

The authors received no financial support for the research, authorship, and/or publication of this paper. The authors have reported that they have no relationships relevant to the contents of this paper to disclose.
