# Supplementary material for: Prognostic Significance of Plasma Lactate Levels in Postcardiotomy Patients
Source: JACC Adv. 2026 Aug 18;5(9):103158. doi: 10.1016/j.jacadv.2026.103158 (PMC13520724; doi:10.1016/j.jacadv.2026.103158)
Supplement: Supplementary file 1 [file mmc1.docx]

**Supplemental Text**

**Text 1: Definitions of the most important variables and SCAI SHOCK classification**

All definitions of the variables are according to the standardized criteria outlined by the XXX Heart Registry (NHR) are used [20]. Key definitions are described here. Left ventricular ejection fraction (LVEF) was categorized as good (LVEF≥50%), mildly reduced (LVEF 41-49%), or moderate (≤40%). Recent myocardial infarction was defined according to established criteria as occurring within 90 days before surgery. A myocardial infarction was diagnosed based on troponin levels more than 10 times the upper reference limit. This had to be accompanied by electrocardiographic, angiographic, or echocardiographic evidence of myocardial injury. In postoperative patients, an additional requirement was a creatine kinase (CK) to CK-MB ratio of 10% or less. Postoperative renal failure was defined by either new renal replacement therapy or doubling of preoperative creatinine to > 177 μmol/L. A rhythm disorder was defined as any de novo atrial or ventricular tachy-arrhythmia requiring intervention. Transient, self-limiting atrial fibrillation without clinical consequences was not included. Respiratory insufficiency was defined as the need for re-intubation.

**Supplemental Tables**

**Table 1. Plasma lactate levels of surviving and the non-surviving patients per postoperative day (one through five)**

| **Plasma lactate level (mmol/L)** | **Survivors** | **Non-survivors** | **Total** |
| --- | --- | --- | --- |
| **POD one** | 1.5 [1.2 -2.2] | 3.1 [1.7-6.3] | 1.6 [1.2-2.2] |
| **POD two** | 1.8 [1.3-2.6] | 3.6 [2.4-8.4] | 1.8 [1.3-2.7] |
| **POD three** | 1.5 [1.1-2.0] | 2.5 [1.8-4.7] | 1.5 [1.1-2.1] |
| **POD four** | 1.3[1.0-1.8] | 2.3 [1.5-4.9] | 1.4 [1.0-1.9] |
| **POD five** | 1.3 [1.0-1.6] | 2.1 [1.4-3.9] | 1.3 [1.0-1.7] |

*Notes* POD: postoperative day

**Table 2. Univariate analysis for elevated plasma lactate levels on postoperative day one**

| **Risk factor** | **OR [95% CI]** | **P-value** |  |
| --- | --- | --- | --- |
| **NYHA class IV** | 2.2 [1.8-2.7] | <0.001 |  |
| **Mitral valve surgery** | 2.0 [1.8-2.3] | <0.001 |  |
| **Three or more procedures** | 1.9 [1.6-2.2] | <0.001 |  |
| **Tricuspid valve surgery, isolated and/or combined** | 1.8 [1.5-2.2] | <0.001 |  |
| **Endocarditis** | 1.8 [1.4-2.2] | <0.001 |  |
| **Previous cardiac surgery** | 1.6 [1.3-1.8] | <0.001 |  |
| **Two procedures** | 1.5 [1.3-1.6] | <0.001 |  |
| **Urgent surgery** | 1.4 [1.3-1.5] | <0.001 |  |
| **Aortic surgery, isolated and/or combined** | 1.4 [1.2-1.6] | <0.001 |  |
| **Poor LVEF** | 1.3 [1.1-1.7] | 0.012 |  |
| **Diabetes mellitus** | 1.3 [1.1-1.5] | <0.001 |  |
| **CCS class IV** | 1.3 [1.0-1.6] | 0.018 |  |
| **Moderate PA pressure** | 1.3 [1.1-1.4] | <0.001 |  |
| **EuroSCORE II (per 4 points)** | 1.3 [1.2-1.3] | <0.001 |  |
| **NYHA class III** | 1.2 [1.1-1.4] | <0.001 |  |
| **Arterial vascular pathology** | 1.2 [1.0-1.4] | 0.009 |  |
| **Cardiopulmonary bypass time (per 30 min)** | 1.2 [1.2-1.2] | <0.001 |  |
| **Recent myocardial infarction** | 1.2 [1.0-1.3] | 0.016 |  |
| **BMI (per 5 kg/m²)** | 1.2 [1.1-1.2] | <0.001 |  |
| **Valve surgery, isolated or combined** | 1.1 [1.0-1.2] | 0.011 |  |
| **Moderate LVEF** | 1.1 [1.0-1.3] | 0.034 |  |
| **Aortic cross-clamp (per 15 min)** | 1.1 [1.1-1.1] | <0.001 |  |
| **Age (per 5 years)** | 1.0 [1.0-1.0] | 0.008 |  |
| **NYHA class I** | 0.9 [0.8-1.0] | 0.056 |  |
| **Aortic valve surgery, isolated or combined** | 0.9 [0.8-1.0] | 0.015 |  |
| **Good LVEF** | 0.9 [0.8-0.9] | 0.003 |  |
| **Normal PA pressure** | 0.8 [0.7-0.9] | <0.001 |  |
| **NYHA class II** | 0.8 [0.7-0.8] | <0.001 |  |
| **Elective surgery** | 0.7 [0.6-0.8] | <0.001 |  |
| **One procedure** | 0.7 [0.6-0.8] | <0.001 |  |

*Notes* NYHA: New Year Heart Association; LVEF: left ventricular ejection fraction; CABG: coronary artery bypass grafting; PA: pulmonary artery

**Table 3.** **Distribution of patients across predefined plasma lactate categories (0-2, 2–5, 5–10, and >10 mmol/L) with corresponding survival and in-hospital mortality per category on postoperative day two**

| **Plasma lactate category** | **Survivors (n=7,899)** | **Non-survivors (n=156)** | **P-value** | **OR [95% CI]** | **P-value** |
| --- | --- | --- | --- | --- | --- |
| **0-2 mmol/L** | 4,347 (99%) | 30 (1%) | <0.001 | 0.2 [0.1-0.3] | <0.001 |
| **2-5 mmol/L** | 3,222 (98%) | 65 (2%) | <0.001 | 1.0 [0.7-1.5] | 0.87 |
| **5-10 mmol/L** | 298 (91%) | 30 (9%) | <0.001 | 6.1 [3.9-9.3] | <0.001 |
| **>10 mmol/L** | 32 (51%) | 31 (49%) | <0.001 | 60.8 [34.7-106.3] | <0.001 |

*Notes* OR: odds ratio; CI: confidence interval

**Table 4.** **Distribution of patients across predefined plasma lactate categories (0-2, 2–5, 5–10, and >10 mmol/L) with corresponding survival and in-hospital mortality per category on postoperative day three**

| **Plasma lactate category** | **Survivors (n=2,693)** | **Non-survivors (n=126)** | **P-value** | **OR [95% CI]** | **P-value** |
| --- | --- | --- | --- | --- | --- |
| **0-2 mmol/L** | 1,948 (98%) | 40 (2%) | <0.001 | 0.2 [0.1-0.3] | <0.001 |
| **2-5 mmol/L** | 705 (93%) | 57 (8%) | <0.001 | 2.3 [1.6-3.4] | <0.001 |
| **5-10 mmol/L** | 38 (73%) | 14 (27%) | <0.001 | 8.7 [4.2-17.0] | <0.001 |
| **>10 mmol/L** | 2 (12%) | 15 (88%) | <0.001 | 180.5 [41.2-1593.5] | <0.001 |

*Notes* OR: odds ratio; CI: confidence interval

**Table 5.** **Distribution of patients across predefined plasma lactate categories (0-2, 2–5, 5–10, and >10 mmol/L) with corresponding survival and in-hospital mortality per category on postoperative day four**

| **Plasma lactate category** | **Survivors (n=1,295)** | **Non-survivors (n=107)** | **P-value** | **OR [95% CI]** | **P-value** |
| --- | --- | --- | --- | --- | --- |
| **0-2 mmol/L** | 1,034 (96%) | 42 (4%) | <0.001 | 0.2 [0.1-0.3] | <0.001 |
| **2-5 mmol/L** | 257 (87%) | 40 (14%) | <0.001 | 2.4 [1.5-3.7] | <0.001 |
| **5-10 mmol/L** | 4 (19%) | 17 (81%) | <0.001 | 60.3 [19.2-252.7] | <0.001 |
| **>10 mmol/L** | 0 | 8 | <0.001 | NA | NA |

*Notes* OR: odds ratio; CI: confidence interval; NA: not available due to 0 survivors in >10 mmol/L category

**Table 6.** **Distribution of patients across predefined plasma lactate categories (0-2, 2–5, 5–10, and >10 mmol/L) with corresponding survival and in-hospital mortality per category on postoperative day five**

| **Plasma lactate category** | **Survivors (n=769)** | **Non-survivors (n=93)** | **P-value** | **OR [95% CI]** | **P-value** |
| --- | --- | --- | --- | --- | --- |
| **0-2 mmol/L** | 652 (94%) | 43 (6%) | <0.001 | 0.2 [0.1-0.2] | <0.001 |
| **2-5 mmol/L** | 107 (76%) | 33 (24%) | <0.001 | 3.4 [2.0-5.6] | <0.001 |
| **5-10 mmol/L** | 7 (54%) | 6 (46%) | <0.001 | 7.5 [2.0-26.6] | 0.001 |
| **>10 mmol/L** | 3 (21%) | 11 (79%) | <0.001 | 33.0 [8.7-193.2] | <0.001 |

*Notes* OR: odds ratio; CI: confidence interval

**Table 7. Receiver operating characteristic curves of plasma lactate cut-offs (≥2, ≥5, and ≥10 mmol/L) for prediction of in-hospital mortality on postoperative day one-five**

| **POD** | **Threshold plasma lactate** | **Specificity** | **Sensitivity** | **AUC** | **P-value** |
| --- | --- | --- | --- | --- | --- |
| **POD 1** | ≥2 | 0.68 | 0.71 | 0.69 | <0.001 |
| **POD 1** | ≥5 | 0.98 | 0.33 | 0.65 | <0.001 |
| **POD 1** | ≥10 | 1.00 | 0.11 | 0.55 | <0.001 |
| **POD 2** | ≥2 | 0.55 | 0.81 | 0.68 | <0.001 |
| **POD 2** | ≥5 | 0.96 | 0.39 | 0.68 | <0.001 |
| **POD 2** | ≥10 | 0.99 | 0.20 | 0.60 | <0.001 |
| **POD 3** | ≥2 | 0.72 | 0.68 | 0.70 | <0.001 |
| **POD 3** | ≥5 | 1.00 | 0.23 | 0.61 | <0.001 |
| **POD 3** | ≥10 | 1.00 | 0.12 | 0.56 | <0.001 |
| **POD 4** | ≥2 | 0.80 | 0.61 | 0.70 | <0.001 |
| **POD 4** | ≥5 | 1.00 | 0.23 | 0.62 | <0.001 |
| **POD 4** | ≥10 | 1.00 | 0.08 | 0.54 | <0.001 |
| **POD 5** | ≥2 | 0.85 | 0.54 | 0.69 | <0.001 |
| **POD 5** | ≥5 | 0.99 | 0.18 | 0.59 | <0.001 |
| **POD 5** | ≥10 | 1.00 | 0.12 | 0.56 | <0.001 |

*Notes* POD: postoperative day; AUC: area under the curve; P‑values were obtained using the Wilcoxon–Mann–Whitney test to assess whether the AUC was significantly greater than 0.50 (better than chance).

**Table 7. Brier scores for plasma lactate, SCAI SHOCK classification and EuroSCORE II to predict in‑hospital mortality across PODs one through five**

| **Postoperative day** | **Model** | **Brier score** |
| --- | --- | --- |
| **One** | Plasma lactate | 0.0161 |
| **One** | SCAI SHOCK | 0.0162 |
| **One** | EuroSCORE II | 0.0166 |
| **Two** | Plasma lactate | 0.0150 |
| **Two** | SCAI SHOCK | 0.0147 |
| **Two** | EuroSCORE II | 0.0160 |
| **Three** | Plasma lactate | 0.0339 |
| **Three** | SCAI SHOCK | 0.0327 |
| **Three** | EuroSCORE II | 0.0365 |
| **Four** | Plasma lactate | 0.0485 |
| **Four** | SCAI SHOCK | 0.0496 |
| **Four** | EuroSCORE II | 0.0584 |
| **Five** | Plasma lactate | 0.0758 |
| **Five** | SCAI SHOCK | 0.0676 |
| **Five** | EuroSCORE II | 0.0781 |

**Supplemental Figures**

**Figure 1. Flowchart of the inclusion and exclusion of the cohort**

*Notes* PCI: percutaneous coronary intervention; ECLS: extracorporeal life support

**Figure** **2**. **Classification of the SCAI SHOCK classification**

Stage A

Hemodynamically stable

Stage B

SBP between 60-90 mm Hg or plasma lactate levels between 2 and 5 mmol/L + no vasopressors and/or inotropes

Stage C

SBP between 60-90 mm Hg + plasma lactate levels between 2 and 5 mmol/L + no vasopressors and/or inotropes

use of 1 vasopressor or inotrope + plasma lactate levels between 2 and 5 mmol/L or SBP < 90 mm Hg

SBP above 90 mm Hg + plasma lactate levels between 5 and 10 mmol/L + no vasopressors and/or inotropes

use of MCS (i.e. ECLS, Impella or IABP)

Stage D

SBP between 60-90 mm Hg + serum lactate between 5 and 10 mmol/L + no vasopressor and/or inotropes

2-5 vasopressors and/or inotropes

SBP between 60-90 mm Hg + serum lactate between 2 and 5 mmol/L + 1 vasopressor or inotrope

Stage E

SBP under 60 mm Hg

serum lactate above 10 mmol/L

≥3 vasopressors and/or inotropes

*Notes* SCAI: Society of Cardiovascular Angiography and Interventions; SBP: systolic blood pressure; MCS: mechanical circulatory support; ECLS: extra corporeal life support; IABP: intra-aortic balloon pump

**Figure** **3**. **Receiver operating characteristic curves of plasma lactate, SCAI SHOCK and EuroSCORE II on postoperative day one**

**Figure** **4**. **Receiver operating characteristic curves of plasma lactate, SCAI SHOCK and EuroSCORE II on postoperative day two**

**Figure** **5**. **Receiver operating characteristic curves of plasma lactate, SCAI SHOCK and EuroSCORE II on postoperative day three**

**Figure** **6**. **Receiver operating characteristic curves of plasma lactate, SCAI SHOCK and EuroSCORE II on postoperative day four**

**Figure** **7**. **Receiver operating characteristic curves of plasma lactate, SCAI SHOCK and EuroSCORE II on postoperative day five**

**Figure** **8**. **Calibration plots for plasma lactate, SCAI SHOCK stage and EuroSCORE II on postoperative day one**

**Figure 9.** **Calibration plots for plasma lactate, SCAI SHOCK stage and EuroSCORE II on postoperative day two**

**Figure** **10**. **Calibration plots for plasma lactate, SCAI SHOCK stage and EuroSCORE II on postoperative day three**

**Figure** **11**. **Calibration plots for plasma lactate, SCAI SHOCK stage and EuroSCORE II on postoperative day four**

**Figure** **12**. **Calibration plots for plasma lactate, SCAI SHOCK stage and EuroSCORE II on postoperative day five**
